# Neurobiology of social reward valuation in adults with a history of anorexia nervosa

**DOI:** 10.1371/journal.pone.0205085

**Published:** 2018-12-04

**Authors:** Maggie M. Sweitzer, Karli K. Watson, Savannah R. Erwin, Amy A. Winecoff, Nandini Datta, Scott Huettel, Michael L. Platt, Nancy L. Zucker

**Affiliations:** 1 Department of Psychiatry & Behavioral Sciences, Duke University School of Medicine, Durham, North Carolina, United States of America; 2 Institute for Cognitive Neuroscience, University of Colorado, Boulder, Colorado, United States of America; 3 Department of Psychology and Neuroscience, Duke University, Durham, North Carolina, United States of America; 4 Medical Scientist Training Program, Duke University School of Medicine, Durham, North Carolina, United States of America; 5 Department of Neuroscience, Perelman School of Medicine, University of Pennsylvania, Philadelphia, Pennsylvania, United States of America; 6 Department of Psychology, University of Pennsylvania, Philadelphia, Pennsylvania, United States of America; 7 Marketing Department, The Wharton School, University of Pennsylvania, Philadelphia, Pennsylvania, United States of America; King's College London, UNITED KINGDOM

## Abstract

**Objective:**

Anorexia nervosa (AN) is a disorder characterized by atypical patterns of reward valuation (e.g. positive valuation of hunger). Atypical reward processing may extend into social domains. If so, such findings would be of prognostic significance as impaired social functioning predicts worse outcome. We explore neural circuits implicated in social reward processing in individuals with a history of AN who are weight-restored relative to controls and examine the effects of illness course on the experience of social value.

**Method:**

20 weight-restored individuals with a history of AN (AN-WR) and 24 healthy control (HC) participants were assessed using fMRI tasks that tapped social reward: smiling faces and full human figures that varied in attractiveness and weight.

**Results:**

AN-WR differed from HC in attractiveness ratings by weight (negatively correlated in AN-WR). While there were no significant differences when viewing smiling faces, viewing full figures resulted in decreased activation in regions implicated in reward valuation (the right caudate) for AN-WR and this region was negatively correlated with a sustained course of the disorder. Exploratory whole brain analyses revealed reduced activation in regions associated with social reward, self-referential processing, and cognitive reappraisal (e.g., medial prefrontal cortex, striatum, and nucleus accumbens) with sustained disorder course.

**Discussion:**

The rewarding value of full body images decreases with a sustained disorder course. This may reflect an extension of atypical reward processing documented in AN-WR, perhaps as a function of starvation dampening visceral motivational signals; the deployment of cognitive strategies that lessen the experience of reward; and/or the nature of the stimuli themselves as provocative of eating disorder symptoms (e.g., thin bodies). These findings did not extend to smiling face stimuli. Advances in technology (e.g., virtual avatars, text messaging) may provide novel means to build relationships, including therapeutic relationships, to support improved social connections without threats to symptom provocation.

## Introduction

One obstacle for the lasting treatment of anorexia nervosa (AN) is identifying sources of reinforcement that compete with the reward value of the drive for thinness [1–3]. AN is characterized as ego-syntonic: individuals with AN will work for, and therefore value, core symptoms that many would find aversive (e.g., hunger) [1, 4]. This clinical presentation may reflect aberrations in reward valuation: those with AN may assign negative valence to stimuli and experiences that typically hold positive value (e.g., food, high fat tastes), assign positive value to stimuli or conditions that are typically aversive (e.g., hunger), and experience punishing stimuli as particularly aversive [3, 5–10].

Distorted reward valuation processes may apply to domains of reward beyond solely gustatory stimuli [11, 12]. Individuals with AN report reduced fun-seeking [13], higher levels of social anhedonia [14], and lower levels of sexual drive [15, 16], all of which may be related to levels of social reward experience. Moreover, weight-restored individuals with AN demonstrated reduced reward value for visual images of people in a laboratory choice task and spent less time looking at the face and eye regions during an eye-tracking task [17].

Assigning value to a stimulus is contingent upon context: a highly palatable food may be experienced as less palatable in a social context in which people are discussing the stigma associated with weight gain [18]. AN typically onsets in early adolescence, a developmental period in which interoceptive states are increasingly elaborated by social contexts [19, 20]. Thus, it is possible that interdependent processes may be contributing to atypical reward-stimulus assignment in AN: suppression of the visceral components of reward experience as a consequence of starvation (i.e., the dampening of visceral volatility that accompanies starvation) and occurrence of starvation during a period of brain development when visceral sensations are increasingly elaborated with social and other contexts [20–22].

One explanation for these observations is the development of atypical functioning in the neural circuits that process rewards, such as the ventral and dorsal striatum and prefrontal cortex. Both the ventral striatum (VS), encompassing the nucleus accumbens, and dorsal striatum (DS), including the caudate and putamen, are heavily interconnected with prefrontal cortical regions, and are critical substrates of reinforcement learning, reward valuation, and motivated behavior [23]. Although both VS and DS are responsive to anticipation and receipt of reward, functional specialization within the striatum has been demonstrated along a ventral to dorsal axis [24]. The VS and vmPFC form the ventral/limbic circuit, which is involved in subjective valuation of reward, prediction error, attributing incentive salience to stimuli and generating emotional responses. The DS, particularly the caudate and anterior putamen, and the dorsal ACC form the dorsal/executive circuit, which is involved in reinforcement learning, planning and monitoring, and integrating reward feedback to motivate behavior and guide decisions [25]. Previous studies of AN have demonstrated aberrant signaling in the VS in response to salient food stimuli [26], likely driven by down-regulation of hypothalamic input to the VS via ACC [27] or other cognitive control regions [28]. Studies of reward responsiveness to non-food stimuli in AN have been equivocal in demonstrating deficits in striatal activation to monetary reward or positive feedback, but a clear pattern has emerged suggesting heightened tracking of prediction error in the VS [29] and increased sensitivity to punishment [12, 25], including social rejection [30], in the caudate. The midline regions of the prefrontal cortex manifest the same ventral to dorsal gradient as the striatum [23]. The vmPFC region is implicated in the summation of value irrespective of reward type [23, 31]. Atypical functioning in the vmPFC would be consistent with the documented aberrancies in reward valuation across multiple domains in AN.

Excessive monitoring of error-related feedback may dampen the experience of socially rewarding stimuli. Extending dorsally, the dACC has been shown to respond to both reward and punishment, and may facilitate vigilance in monitoring for behaviorally-relevant feedback [32]. Increased dACC activation has been associated with increased feedback learning following negative feedback among AN patients relative to HC, correlating with perfectionism [33]. Conversely, another study found that AN patients had higher error rates and exhibited decreased activation throughout reward-related corticostriatal circuits including VS, ACC, and thalamus during a target-detection task requiring shifts in behavioral response compared with HC [34]. Thus, the neurobehavioral phenotype of AN presents a paradox of increased responsiveness to negative feedback characterized by increased striatal response to punishments and heightened dACC activation, but decreased cognitive flexibility in adapting to changing circumstances characterized by hypoactivation of corticostriatal circuits. This paradox may suggest a role for diminished dACC involvement in responding to reward contingencies and positive social stimuli to flexibly guide and motivate behavior, rather than relying on higher-order cognitive control and rigid rules.

Combined, these findings suggest several hypotheses as to if and why the experience of social reward may be dampened in AN. First, changes in reward experience with a sustained starvation, such as the dampening of visceral sensations that constitute emotional experience and self-awareness, may broadly impact all forms of reward including social reward. Second, the excessive error monitoring and inflexible cognitive strategies may be particularly ill-suited to manage complex social interactions resulting in less reinforcement. To date, there is evidence to support both of these hypotheses.

At the nexus between vmPFC and dACC, the medial prefrontal cortex (mPFC) is central to the emotional processing of social reward. Social reward processing involves higher order neural representations of future goals and self-identity that are dependent on prefrontal involvement—particularly reciprocal connections between the mPFC, VS, and related regions [35, 36]. Consistent with behavioral studies indicating higher levels of social anhedonia in AN, a recent study found decreased mPFC activation to social acceptance among AN patients compared with healthy controls [30]. Furthermore, both acute and weight-restored women with AN exhibited less mPFC activation during a task involving self-relevant cognitions [37], suggesting that both self-referential and social processing, mediated by mPFC, may be persistently altered in AN.

However, social reward may also become compromised because of unique features inherent to the diagnosis of AN such as the relentless drive for thinness, which is measured by their own personal evaluations of body weight, and their weight in relation to the thinness of others. Other people can be viewed as sources of competition or threats to status and thus activate weight loss goals, and these goals would dampen more affiliative positive emotions towards others. As the disorder progresses, social avoidance becomes more habitual and internal states such as hunger or emotional experience become muted or suppressed via cognitive strategies. Thus, there could be both less activation in bottom-up inputs and more habitual patterns of response, resulting in decreased conflict-related activation and less activation in key motivational regions over time. This pattern would also suggest that the bodies of other individuals may influence the experience of social reward differentially than just viewing non-threatening social stimuli, such as faces.

To investigate social reward processing, we employed functional magnetic resonance imaging (fMRI) to examine neural activity and subjective evaluations of full human images (bodies with faces) of varying weight status (e.g., emaciated, normal weight, and overweight bodies with faces) and smiling faces only in a sample of adult women with a history of AN who were weight-restored (anorexia nervosa weight-restored: AN-WR) as well as a typically-developing healthy control group (HC). Both faces and bodies provide a range of socially relevant information, and induce motivated visual information gathering in the typical individual [38]. Changes in reward valuation were investigated as a function of disorder course. We hypothesized that individuals in the AN-WR group will demonstrate diminished activation in reward-related regions, including the medial prefrontal cortex (mPFC) and striatum, while viewing social stimuli whether full bodies or smiling faces; ultimately reflecting a generalization of diminished reward valuation beyond disorder-related domains. Additionally, we hypothesized that individuals in the AN-WR group, despite weight-restoration, will differ in the subjective valuation of attractiveness as a function of weight category relative to control participants (cf. [17]). Lastly, we predict that impaired social reward valuation will be worse in individuals with a more stable disorder course, reflecting a process of assigning value that becomes exaggerated with disorder progression, a hypothesis consistent with the model of neuroprogressive changes with illness duration proposed by Treasure, Stein, and Maguire [39].

## Methods

### Participants

To be eligible, control participants could not have a history of an eating disorder, did not meet current diagnostic criteria for a psychiatric disorder, and were free of current symptoms of psychosis, substance use, or a neurological disorder. Clinical participants were required to have a prior diagnosis of AN in accordance with DSM-5 criteria [40] and to have maintained a healthy weight for at least six months. All participants completed an online interview detailing the onset, course, and duration of each eating disorder symptom that included portions of the Structured Interview for Anorexia and Bulimia [41] and the complete Eating Disorder Examination-Questionnaire (EDE-Q, Version 6, see below)[42]. Healthy control participants were matched to clinical participants on age, race, education, and medication status. Data from one HC participant and two AN participants were discarded due to an error in the output file from the fMRI scanning session and excessive head motion, respectively. Two other HC participants’ data were discarded due to reported recreational drug use. This resulted in a final sample of 20 AN-WR participants and 24 HC participants. The majority of the sample had been weight-restored for at least three years.

### Survey and behavioral measures

#### Eating Disorders Examination Questionnaire, [EDE-Q; 42]

The EDE-Q is a 28-item self-report questionnaire that provides a continuous measure of eating disorder symptomatology and is broken into four subscales: restraint, eating concern, shape concern, and weight concern. The EDE-Q has been shown to have good psychometric properties [43, 44].

#### Disease and symptom course ratings

Individuals completed a detailed survey on the history of their eating disorder symptoms and prior eating disorder diagnoses. First, individuals were asked to provide a narrative account of their illness history due to the limited richness of information provided by quantitative indices of illness history. Individuals reported on the time from today backwards since they have engaged in or experienced each of the following symptoms (if at all): binge eating; self-induced vomiting; maintaining an unhealthy low weight; laxative abuse; diuretic abuse; steroid abuse; diet pill abuse; driven, compulsive exercise; and other unhealthy weight control strategies (Table 1). AN often has a relapsing course and, as with any mental illness, periods in which symptoms are relatively worse. We assumed that the worst period of one’s illness would be one in which individuals would have a relatively vivid recollection and would help characterize the nature of their disorder history. Thus, we asked individuals to describe the symptoms that characterized their worst episode, focusing on age of onset and offset of that worst episode; the lowest and highest weight during that episode; and then asked about the presence and frequency of each symptom during their worst episode. Finally, we asked individuals to consider the entire course of their disorder–from initial onset to the time when they no longer had an eating disorder and characterize the course of particular symptoms. After individuals had gone through this process of delineating the course of each symptom, we then ask them to reflect on the course of their disorder. The stability of disease course was assessed using questions adapted from the Structured Interview of Anorexia and Bulimia [41]. Participants could respond using a 0–4 response where: 0 indicated no eating disorder, 1 indicated that during the course of their disorder, symptoms occurred up to 30% of time (or less) (or a very intermittent course), 2 indicated that symptoms were present about 30–60% of the time (a moderately intermittent course), 3 indicated that symptoms were present roughly 60–90% of the time (a relatively stable course), and 4 indicated that symptoms were present roughly 90–100% of the time (a stable course). Note that these course descriptions are provided to aid the reader but were not provided to the study participants. Responses were aided via the use of graphic depictions of illness course (S1 Fig). Length of illness was used as an index of disorder severity.

**Table 1:** Demographic and eating disorder characteristics by group.

	HC	AN-WR
**Demographics**	n (%)	
Race		
Caucasian	18 (75.0%)	15 (75.0%)
African American	2 (8.3%)	1 (5.0%)
Asian	3 (12.5%)	2 (10.0%)
Indian	0 (0%)	1 (5.0%)
Native American	1 (4.2%)	0 (0.0%)
Not Reported	0 (0%)	1 (5.0%)
	Mean (SD)	
Age (years)	24.50 (3.65)	22.50 (3.65)
Education (years)	15.54 (1.61)	15.37 (2.50)
**Comorbidity and Psychotropic Medication**	n (%)	
Non-eating disorder Mental Illness Diagnosis	2 (8.3%)	6 (30.0%)
Current Psychoactive Medication Use	0 (0.0%)	3 (15.0%)
**Eating Disorder Examination Questionnaire (EDE-Q)**^**#**^	Mean (SD)	
Restraint*	0.45 (0.84)	1.04 (0.96)
Eating Concern*	0.13 (0.31)	0.46 (0.60)
Shape Concern*	0.63 (0.78)	1.48 (1.53)
Weight Concern*	0.40 (0.53)	1.29 (1.12)
Global*	0.40 (0.56)	1.07 (0.94)

Note: Group summaries are based on 20 participants with a prior diagnosis of anorexia nervosa and 24, sex, race, and age-matched participants.

*signifies *p* < 0.05

^#^For participants who completed the EDE-Q and reported BMI at two separate time points, values were compiled from the survey completed with the shortest time delay to that participant’s scan date. Average delay between EDE-Q completion/BMI report and scan was 47.92 days (SD = 38.82 days, Range = 2.32–165.63 days).

### Procedure

Prior to testing, participants completed an online battery of survey measures of emotional, interpersonal, and social functioning. Participants provided informed consent for a protocol approved by Duke University Medical Center’s Institutional Review Board and were paid $30 per hour for their time. All participants completed the experimental protocol over three or four testing days. On Day One, participants were consented and familiarized with the fMRI task. On Day Two, participants underwent fMRI scanning. During the final session, participants were shown all scanner images and were asked to rate the attractiveness of face and body images, and the weight of body images, using a 9-point Likert scale.

#### Social fMRI task

*Image Selection*. Three classes of stimuli were displayed to participants in the scanner: full body images with faces visible, images of faces, and scrambled images of the stimuli as a visual control. To select images for use in the fMRI portion of the experiment, we first ran a pilot study in which college-aged female participants (n = 17) rated 480 body images and 268 face images for weight and attractiveness, each on a 1 to 10 Likert scale. Images were selected based on weight and attractiveness ratings to maximize variance. All images depicted women approximately 18 to 30 years of age and were drawn from publicly available Internet sources (see S1 File).

Of the 480 body images scored, 144 images were selected for use in the fMRI portion of our experiment. In order to create a stimulus set with a wide range of body weights, we chose images depicting the 48 heaviest, 48 intermediate, and the 48 thinnest individuals (based on participant ratings). The images of intermediate body weight were selected to minimize variance along the attractiveness dimension.

**Fig 1:**
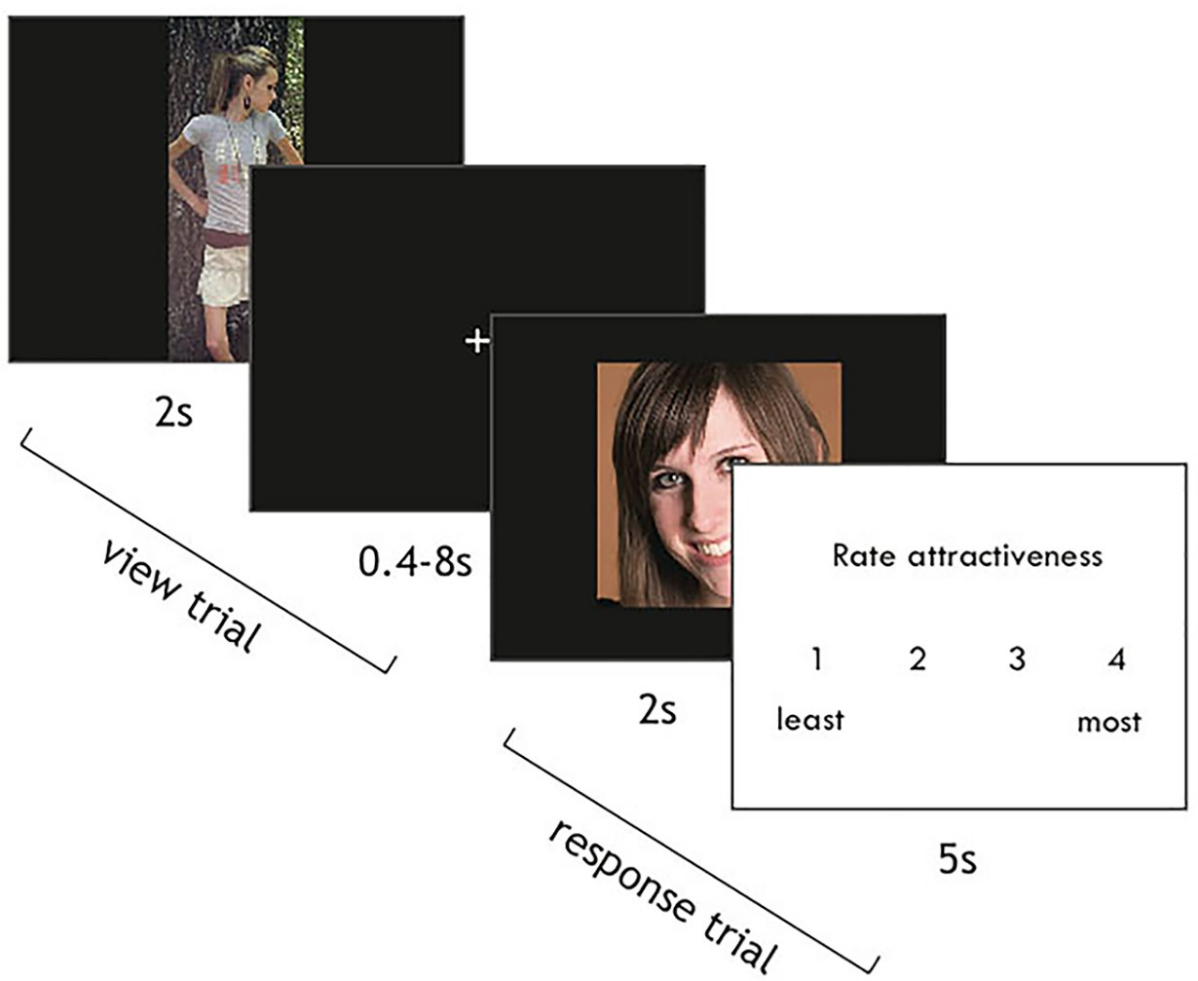
fMRI task paradigm. Participants viewed images of faces, bodies with faces, and scrambled images for 2s each. On most trials, image presentation was followed by a variable length inter-trial interval. On a subset of trials, participants were given 5s to rate the attractiveness of each image using a button box.

*fMRI Task*. The scanner task consisted of 6 functional runs with 72 trials each. We used a mixed blocked/event-related design such that 8 images from each trial type (faces, bodies, scrambled) were presented within each block. At the start of each trial, an image was presented onscreen for 2s. To ensure that participants were actively engaged in the task, on 12 trials per run participants were given 5s to rate the attractiveness of the image on a scale of one to four using a button box (Fig 1). Presentation order was pseudorandomized such that one of ten timing templates designed to maximize model efficiency was selected from a randomized list. Inter-trial intervals were drawn from an exponential distribution with a minimum of 2s and a maximum of 12s. Images were displayed using Neurobehavioral Systems’ Presentation software.

#### fMRI acquisition, preprocessing and first-level modeling

Magnetic resonance images were acquired on a General Electric 3T scanner using a spiral-in pulse sequence. We utilized standard imaging parameters (TR = 1.58s, TE = 30ms, FOV = 243mm, flip angle = 70°, voxel size = 3.8 x 3.8 x 3.8 mm, 37 axial slices collected parallel to the AC-PC plane). To allow for the stabilization of the fMRI signal, the first 5 volumes of each run were discarded. Due to a programming error, runs for the first ten participants contained variable numbers of volumes from 250 to 294. Data acquired for the rest of the participants contained 278 volumes of functional data per run. High-resolution inversion-recovery prepared SPGR structural images were collected for each participant to aid in anatomical registration and normalization (TR = 7.2ms, TE = 2.9ms, FOV = 256mm, flip angle = 12°, voxel size = 1 x 1 x 1mm).

Preprocessing steps were performed in FSL 4.1.8 and included brain extraction using BET, a high pass filter (>100s), spatial smoothing using a 6mm full-width half maximum Gaussian kernel, and motion correction using MCFLIRT. Motion correction occurred with respect to the middle volume of each run. FMRI data were normalized using transforms from each participant’s own high resolution anatomical scan (6 degrees of freedom) and a Montreal Neurological Institute (MNI) anatomical template (12 degrees of freedom) using FLIRT.

Imaging data were analyzed using a general linear model in FSL, FEAT [45]. First-level models included regressors for each of our image types (i.e., faces, bodies, scrambled images) for the 2s presentation period, as well as a nuisance regressor for the response period during trials in which participants made an attractiveness rating. Stimulus onset times were convolved with a standard double-gamma hemodynamic response function, and were corrected for local autocorrelation within a single run [46]. Individual participant effects were estimated for each run using a fixed effects model, and contrast images were created comparing faces > scrambled and bodies > scrambled images. At the second level, a fixed-effects model was used to combine data from all runs within a single participant. Runs with motion exceeding one voxel size in translation or rotation were excluded from second-level models. Two participants with excessive head motion on > 2 runs were excluded from analyses.

#### fMRI and behavioral analysis

*Behavioral Data*. Behavioral data were analyzed using R Studio Version 0.98.1091 and IBM SPSS Statistics Version 24.0. Analyses of EDE-Q scores and demographic characteristics were performed using two-tailed unpaired *t*-tests including group status as the independent variable. All EDE-Q subscale scores were analyzed separately. Weight and attractiveness ratings were analyzed using a linear mixed-effects model (lme function in R) with a maximum likelihood estimation method. Ratings analyses included participant as a random effect and group as a fixed effect. All reported statistics represent Type-III sums of squares.

**Fig 2:**
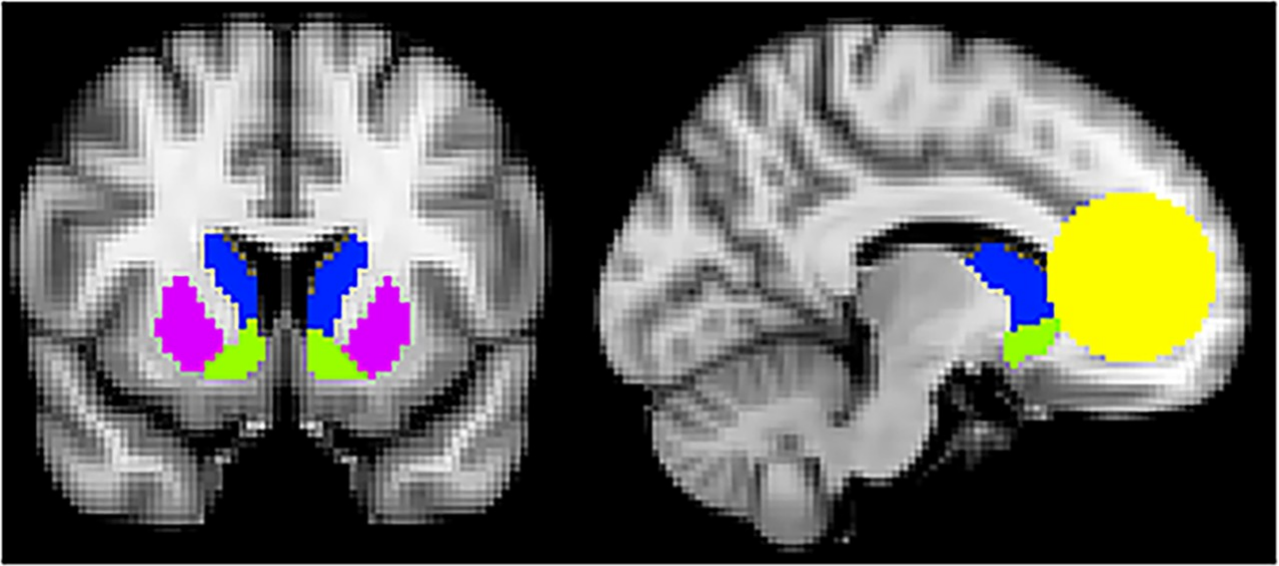
Regions of interest mask. Regions of interest were evaluated within an *a priori* mask encompassing caudate (blue), ventral striatum (green), putamen (purple), and medial prefrontal cortex (yellow).

*fMRI Data*. Third-level group models were analyzed using FSL’s FLAME1 mixed-effects model. Given our interest in reward processing within the striatum and mPFC, primary analyses were conducted using an *a priori* mask of these regions of interest. The striatum was defined using the FSL-Oxford striatal atlas [47] supplied with FSL, which provides contiguous coverage of the ventral striatum, caudate and putamen. MPFC was defined based on our previous neuroimaging work [48] and previous studies of social cognition and reward processing [49, 50] as a sphere of 25 mm radius encompassing medial regions of Brodmann Area (BA) 9 and 10 and BA32, as well as superior portions of BA14 (Fig 2). Differences between AN-WR and HC groups were examined using independent-samples t-tests for each of our primary contrasts (Faces > Scrambled and Bodies > Scrambled). Age was included as a nuisance regressor orthogonalized to each group’s mean. In addition to overall group differences, we tested for the effects of disease course on patterns of neural activation associated with each contrast by creating separate models using clinical participants’ scores on the course question of the clinical interview. To isolate activation correlated with disorder course, the score on this measure was orthogonalized with respect to the group’s mean. Finally, exploratory whole-brain analyses were conducted to examine areas of activation outside of hypothesized ROIs. All reported results survived correction for multiple comparisons (individual voxel threshold *z* > 3.1 for p < 0.001; cluster-corrected significance threshold: *p* < 0.05) within the *a priori* ROI mask or across the whole-brain, as indicated. All coordinates are reported in MNI space.

## Results

### Sample characteristics

Demographic characteristics of the final sample are presented in Table 1. Because this study began when DSM-IV criteria were still in place, DSM-5 diagnoses [40] were inferred based on DSM-IV diagnoses, symptom endorsement, and descriptive illness histories. Under DSM-IV guidelines, 13 clinical participants endorsed a history of anorexia nervosa, six clinical participants endorsed a history of subthreshold anorexia nervosa (lost too much weight, was fearful of weight gain, felt my weight ruled my life) with irregular menstruation or amenorrhea, and one clinical participant endorsed subthreshold anorexia nervosa with regular menstruation. With the changes made in DSM-5 [40], all 20 clinical participants would meet criteria for AN. Participants in the AN-WR group significantly differed from those in the HC group on each of the EDE-Q subscales (Restraint: *t*(42) = 2.17, *p* = .036; Eating Concern: *t*(27) = 2.22, *p* = 0.035; Shape Concern: *t*(27) = 2.24, *p* = 0.034; Weight Concern: *t*(26) = 3.26, *p* = 0.003; Global: *t*(30) = 2.77, *p* = 0.010), but did not differ on age (*t*(42) = -1.81, *p* = 0.077), BMI (*t*(30) = -1.535, *p* = .135) or education (*t*(41) = -0.28, *p* = 0.785). Levene’s test indicated unequal variances for between-group comparisons of EDE-Q Eating Concern (*F* = 10.894, *p* = .002), Shape Concern (*F* = 9.736, *p* = .003), Weight Concern (*F* = 12.190, *p* = .001), and Global (*F* = 7.575, *p* = .009) subscales as well as BMI (*F* = 4.909, *p* = .032); degrees of freedom were adjusted accordingly. Mean EDE-Q subscale scores are consistent with another study investigating adult AN following weight restoration [51] and are 1< SD from community norms [52]. None of the HC group and only 15.0% (n = 3) of the AN-WR group were currently taking psychoactive medications (fluoxetine, *n =* 2; Pristiq and clonazepam, *n =* 1). Two (8.3%) participants in the HC group and 6 (30.0%) participants in the AN-WR group had been diagnosed with a mental illness other than an eating disorder by a healthcare provider. These included major depressive disorder (*n =* 6; obsessive-compulsive disorder, *n =* 1; social anxiety disorder with major depressive disorder, *n =* 1). Clinical participants had an average age of onset during adolescence (*M* = 14.10, range = 11–18), and an average duration of 4.45 (range = 1–13) years. With an average self-reported illness course rating of 3.05 (range = 1–4), 80% of participants in the AN-WR group said that, during the course of their eating disorder, symptoms were present over 60% of the time. The average lowest BMI for the AN-WR group was 16.46 (range = 13.7–18.3). Of participants in the AN-WR group, 22.7% never received treatment for their eating disorder (Table 2).

**Table 2:** Eating disorder characteristics of participants with a history of anorexia nervosa.

Eating Disorder Characteristics					Mean (SD)			
Clinical Impairment Assessment Global Score (0–48)*					1.54 (1.96)		7.30 (9.25)	
Current BMI^#^ (17.4–38.8)					22.48 (4.54)		20.95 (1.67)	
Lowest BMI (13.7–18.3)							16.46 (1.64)	
Age of Onset (11–18)							14.10 (2.02)	
Duration (years; 1–13)							4.45 (3.61)	
					n (%)			
Course^&^								
Symptoms present ≤ 30% of the time							2 (10.0%)	
Symptoms present 30–60% of the time							2 (10.0%)	
Symptoms present 60–90% of the time							9 (45.0%)	
Symptoms present 90–100% of the time							7 (35.0%)	
Treatment Latency^+^								
Less than 6 months							1 (5.0%)	
6–12 months							7 (35.0%)	
13 months-3 years							4 (20.0%)	
More than 3 years							3 (15.0%)	
I never received treatment							5 (25.0%)	
**Symptom Abstinence**^**^**^								
n (%)	Never	> 5 years	> 2 years	> 1 year	< 1 year	< 6 months	< 1 month	Still struggling
Binge Eating	9 (45%)	2 (10%)	2 (10%)	2 (10%)	2 (10%)	0 (0%)	0 (0%)	1 (5%)
Self-Induced Vomiting	10 (50%)	2 (10%)	5 (25%)	1 (5%)	0 (0%)	0 (0%)	0 (0%)	0 (0%)
Unhealthy Low Weight	1 (5%)	4 (20%)	9 (45%)	4 (20%)	0 (0%)	0 (0%)	0 (0%)	0 (0%)
Driven Exercise	4 (20%)	3 (15%)	4 (20%)	3 (15%)	1 (5%)	0 (0%)	0 (0%)	3 (15%)
Laxative Abuse	11 (55%)	2 (10%)	3 (15%)	2 (10%)	0 (0%)	0 (0%)	0 (0%)	0 (0%)
Diet Pill Abuse	10 (50%)	0 (0%)	0 (0%)	1 (5%)	1 (5%)	0 (0%)	0 (0%)	0 (0%)
Diuretic Abuse	10 (50%)	0 (0%)	1 (5%)	1 (5%)	0 (0%)	0 (0%)	0 (0%)	0 (0%)
Steroid Abuse	13 (65%)	0 (0%)	0 (0%)	0 (0%)	0 (0%)	0 (0%)	0 (0%)	0 (0%)
Other	6 (30%)	1 (5%)	1 (5%)	1 (5%)	1 (5%)	0 (0%)	0 (0%)	1 (5%)

*signifies *p* < 0.05

^&^Illness course ratings were based on the time between first onset of symptoms and when participants considered themselves to have “stopped having an eating disorder.”

^+^Treatment Latency was defined as time between onset of symptoms and initiation of treatment.

^^^Symptom Abstinence was in response to the question: “From today backwards, how long has it been since you engaged in the following symptom for at least a few times a week?”

### Image rating results

We first examined whether there were main effects of group on the weight or attractiveness ratings of bodies or the attractiveness ratings of faces. Groups did not differ in their attractiveness ratings of faces (*t*(37) = 0.77, *p* = .44), attractiveness ratings of bodies (*t*(37) = -0.72, *p* = .48), or in their weight ratings of bodies (*t*(35) = -0.92, *p* = .36).

Secondly, we tested whether attractiveness ratings varied as a function of weight status of the images, group, or both. There were significant main effects of attractiveness (*t*(5278) = -6.55, *p* < .0001) and group (*t*(35) = -5.88, *p* < .0001); however, these main effects were qualified by a group by attractiveness rating interaction (*t*(5278) = 9.35, *p* < .001) wherein HC participants showed an overall positive association between weight and attractiveness (*t*(2852) = 6.94, *p* < .001) and AN-WR participants showed an overall negative association (*t*(2426) = -6.32, *p* < .001). Next, we divided images down into quartiles based on the weight ratings of healthy controls (underweight, low-normal, high-normal, overweight) and tested whether groups differed in their attractiveness ratings within weight categories. There was a significant group by weight category interaction whereby AN-WR participants rated low-normal weight bodies as more attractive than HC participants (*t*(35) = -2.18, *p* = .04; AN: *M* = 5.85, HC: *M* = 5.13), but groups did not differ in any other category (underweight: *t*(35) = -1.53, *p* = .14; high-normal: *t*(35) = 0.63, *p* = .53; overweight: *t*(35) = 0.91, *p* = .37).

### fMRI region of interest results

#### Group differences between task conditions

**Fig 3:**
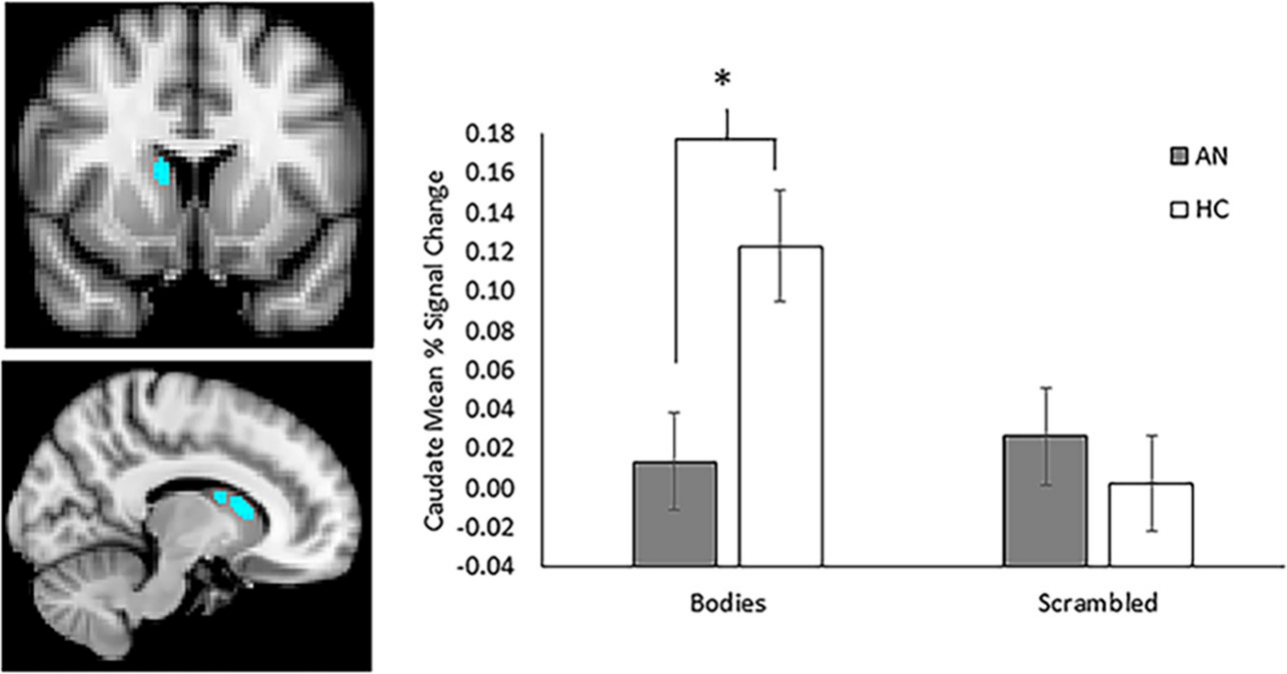
Group contrast comparing activation of bodies relative to scrambled images. Control participants demonstrated increased activation in the right caudate relative to participants with a history of anorexia who were weight restored when viewing whole body images relative to scrambled images. Blocks of body images where collapsed across weight and attractiveness categories. Slices are shown at Y = 10 (top left) and X = 10 (bottom left). Mean percent BOLD signal change extracted from functional cluster in the right caudate for bodies and scrambled images, plotted against group (right panel). Control participants exhibited significantly increased activation to bodies relative to those with history of anorexia, whereas activation to scrambled images did not differ between groups. Error bars represent standard error of the mean.

For the HC group, significant clusters of activation were observed in the mPFC and throughout the striatum including bilateral caudate, putamen, and accumbens for the contrast of Bodies > Scrambled images (Table 3). Similar activation was observed in the mPFC for the AN group, but not in the striatum. Direct contrasts between groups for the Bodies > Scrambled contrast revealed significantly greater activation in the right dorsal caudate for the HC group relative to the AN group (Fig 3). No activation was seen within ROIs for either group for the Faces > Scrambled contrast, and there were no differences between groups.

**Table 3:** Activation to image type by group within mPFC and striatal regions of interest.

Bodies > Scrambled					
**Healthy Controls**	*X*	*y*	*z*	Z-max	voxels
R caudate, accumbens, putamen	10	14	8	5.31	493
Medial superior frontal gyrus	4	56	28	5.12	448
L caudate, accumbens, putamen	-8	10	0	5.14	357
**Anorexia Nervosa-Weight Restored**					
Medial superior frontal gyrus	-6	58	26	5.1	243
**HC > Anorexia Nervosa-Weight-Restored**					
R caudate	12	12	12	3.73	78

mPFC = Medial prefrontal cortex; R = right hemisphere; L = left hemisphere; HC = healthy controls; AN-WR = anorexia nervosa weight restored

Coordinates are in MNI space.

To further explore the pattern of activation within the caudate, mean percent BOLD signal change values were extracted for each image type (Bodies and Scrambled) from the functional cluster using FSL’s featquery. Group means for each task condition, averaged across runs, are presented in Fig 3. Post-hoc analysis indicated that activation to Bodies was significantly greater for HC compared with AN (*t =* 2.85, *p =* 0.007), whereas there was no difference between groups for Scrambled images.

**Individual differences in disorder course**

**Fig 4:**
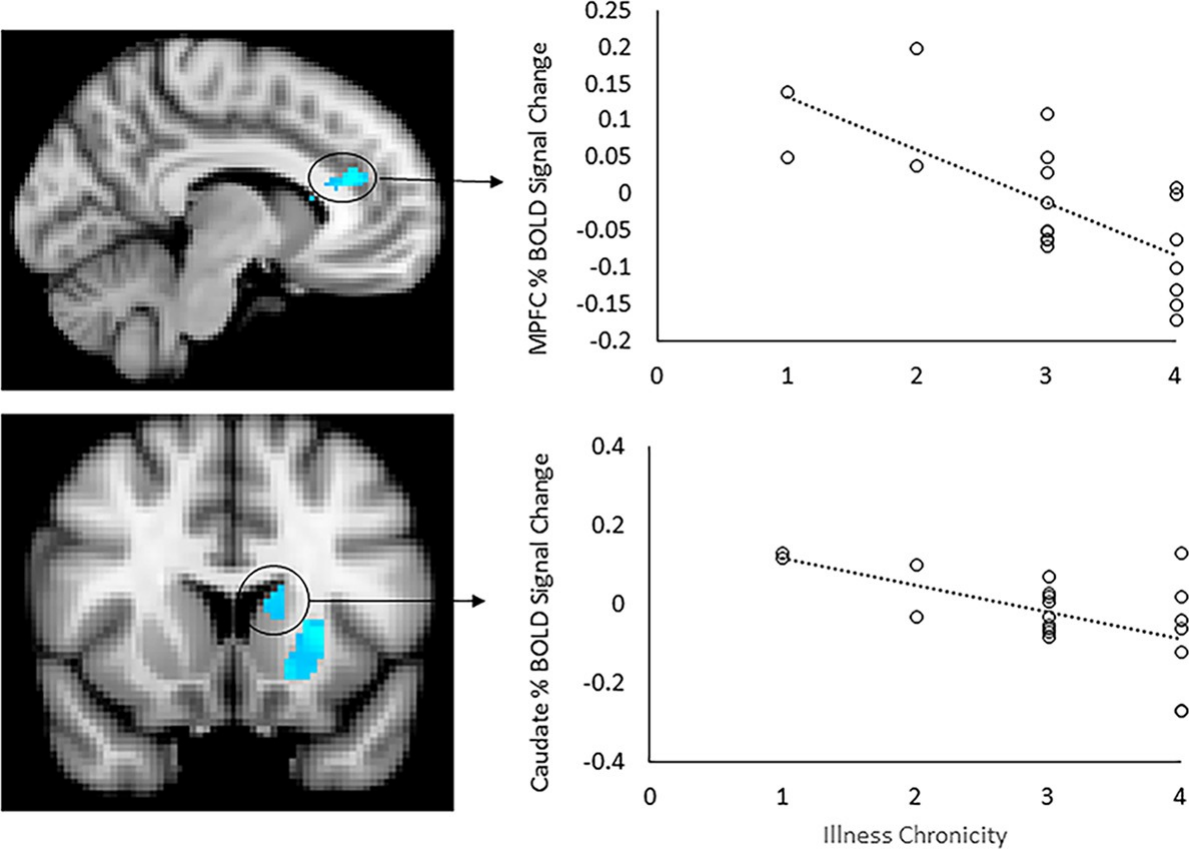
Activation as a function of illness course. Patterns of activation were compared based on the course of illness as collapsed into 4 levels (higher levels indicate more stable illness course). With increased duration of illness, there was decreased activation in the medial prefrontal cortex, bilateral caudate and left putamen and accumbens in the contrast of whole body images > scrambled images (left panel). Slices are shown at X = -8 (top left) and Y = 14 (bottom left). Scatterplots represent mean percent BOLD signal change extracted from significant functional clusters in the mPFC (top right) and left caudate (bottom right), plotted against illness course scores.

Within AN-WR participants, we also examined whether brain activation differed as a function of disorder course. For the contrast of bodies versus scrambled images, illness course was inversely associated with activation in the bilateral caudate and mPFC, and in the left putamen and accumbens. Thus, women with more stable illness course showed decreased activation to bodies of varying weights in reward-related brain regions (Fig 4, Table 4).

**Table 4:** Regions of activation associated with illness course* within mPFC and striatal regions of interest.

Bodies > Scrambled					
**Negative Correlation with Course**	*X*	*y*	*Z*	Z-max	voxels
L mPFC, L caudate	-8	34	22	4.63	262
R mPFC, R caudate	6	34	22	4.12	255
L putamen, accumbens	-26	12	4	4.38	163

*Course is based on an item adapted from the Structured Interview of Anorexia and Bulimia. Individuals are asked to characterize the course of their disorder from the point at which they were first diagnosed with an eating disorder to the period in which they no longer were diagnosed with an eating disorder. Descriptions of four options for disease course were aided with graphic depictions (S1 Fig).

L = Left hemisphere; R = right hemisphere; mPFC = medial prefrontal cortex

Coordinates are in MNI space.

### fMRI exploratory whole-brain results

Main effects of Bodies > Scrambled images and Faces > Scrambled images for each group are presented in S1 Table. For both HC and AN-WR groups, the contrast of face images versus scrambled images revealed widespread activation throughout the inferior division of the lateral occipital cortex and fusiform gyrus, and subcortical regions including bilateral amygdala and hippocampus. Subcortical activation extended to the thalamus and a small portion of the nucleus accumbens for the HC group. For the contrast of bodies versus scrambled images, both groups showed robust activations in the occipital and fusiform cortex, brain stem, bilateral amygdala and parahippocampal gyrus, right pre-central and post-central gyrus, and frontal regions including dorsomedial frontal cortex and right inferior frontal gyrus. Additional activation was observed for the HC group in the paracingulate, lateral orbitofrontal cortex, and throughout the caudate and putamen.

**Fig 5:**
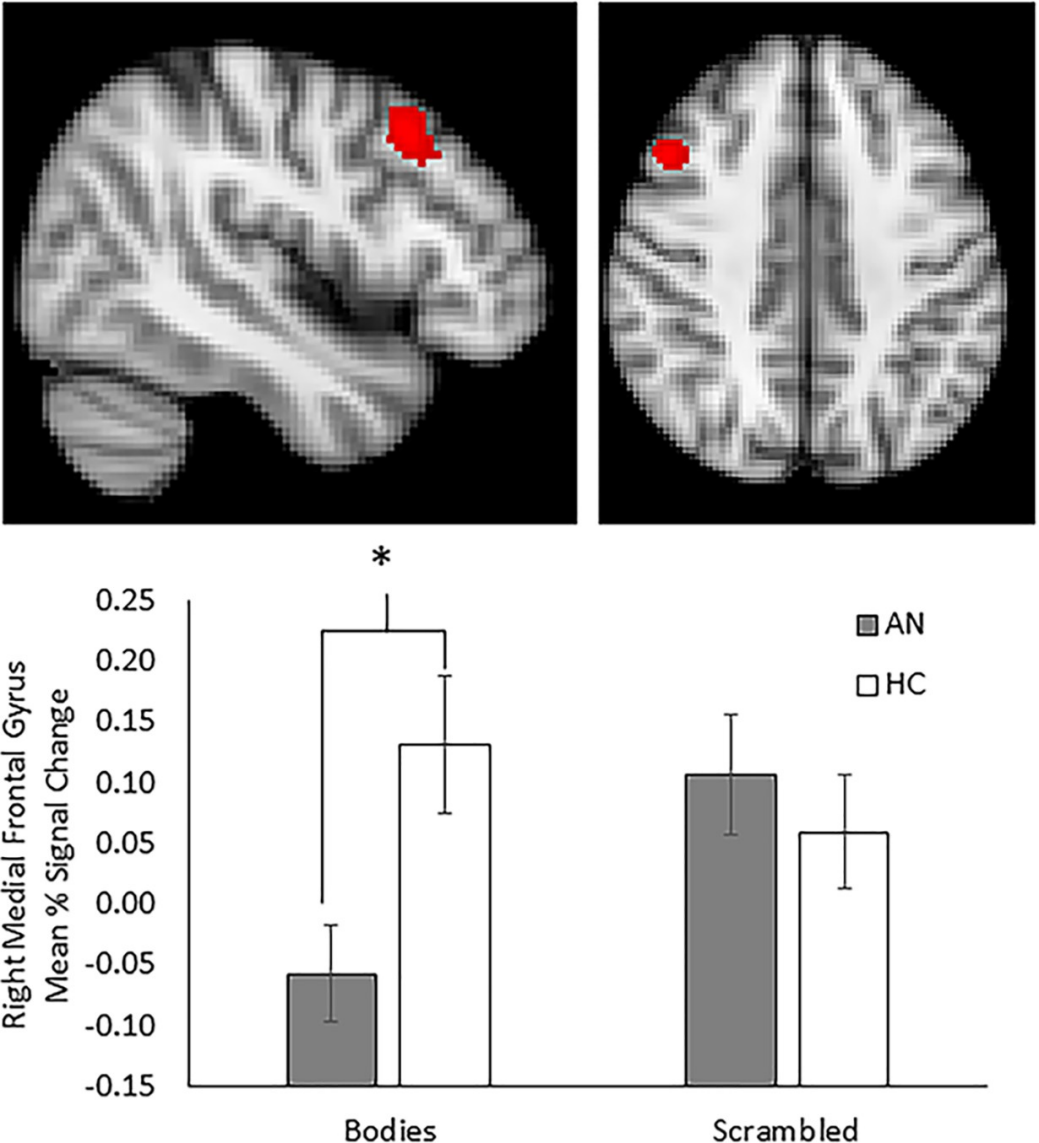
Whole-brain group contrast comparing activation of bodies relative to scrambled images. Weight restored participants with a history of anorexia demonstrated decreased activation in the right middle frontal gyrus relative to controls when viewing whole body images relative to scrambled images. Slices are shown at X = 46 (top left) and Z = 42 (top right). Mean percent BOLD signal change extracted from functional cluster in the right middle frontal gyrus for bodies and scrambled images, plotted against group (bottom panel). Participants with a history of anorexia exhibited significantly decreased activation to bodies relative to control participants, whereas activation to scrambled images did not differ between groups. Error bars represent standard error of the mean.

No group differences were observed for the contrast of Faces > Scrambled images. For Bodies > Scrambled, greater activation was observed for HC compared with AN-WR in a small cluster in the right middle frontal gyrus (183 voxels; Fig 5). Greater activation among HC relative to AN-WR seen in the right caudate with ROI analysis did not survive whole-brain correction. Mean percent signal change extracted from the medial frontal gyrus (MFG) revealed significantly decreased MFG activation for AN-WR compared with HC in response to images of Bodies (*t =* 2.65, *p <* 0.05), whereas response to scrambled images did not differ between the two groups.

**Fig 6:**
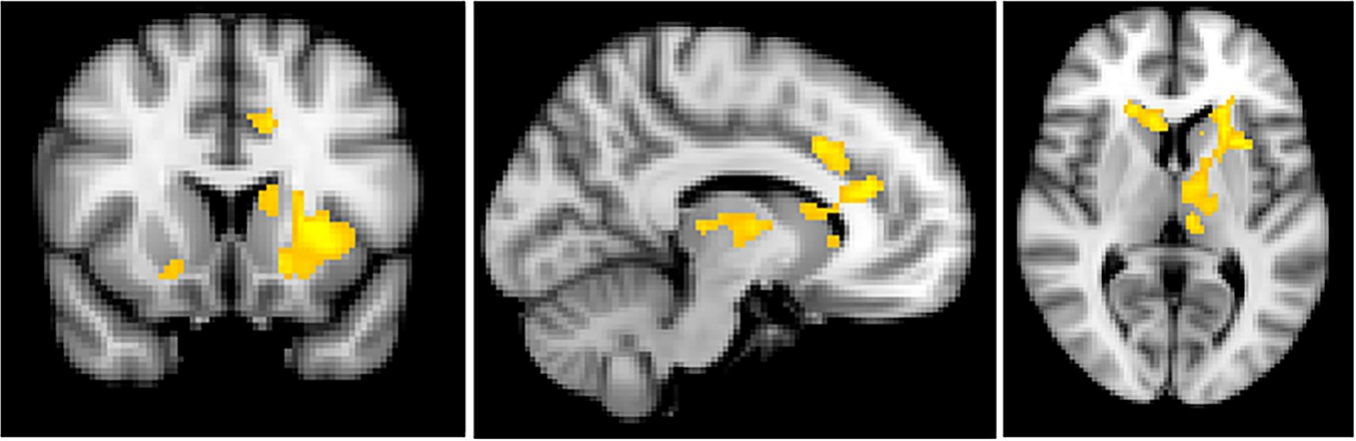
Whole brain activation as a function of illness course. Patterns of activation were compared based on the course of illness as collapsed into 4 levels (higher levels indicate more stable illness course). With increased duration of illness, there was decreased activation in the medial prefrontal cortex and dorsal anterior cingulate cortex, bilateral caudate and putamen, left thalamus and insula, and cerebellum in the contrast of whole body images > scrambled images. Slices are shown at Y = 14 (left), X = -10 (middle), and Z = 6 (right).

When examining associations with illness course across the whole-brain, a negative association was observed between illness course and activation to the Faces > Scrambled contrast in the right middle/inferior frontal gyrus (194 voxels; peak voxel at *x =* 42, *y =* 40, *z =* 6; *Z*_*max*_ *=* 4.28). For the Bodies > Scrambled contrast, a similar pattern of activation was observed as was seen with the ROI analyses. More stable illness course was associated with decreased activation in the mPFC and left caudate/putamen, with additional activation extending to the left insula, right caudate and putamen, left thalamus, and cerebellum (Fig 6; Table 5).

**Table 5:** Regions of activation associated with illness course* across the whole brain.

**Faces > Scrambled**					
**Negative Correlation w/ Course**					
R Middle/inferior frontal gyrus	42	40	6	4.28	194
**Bodies > Scrambled**					
**Negative Correlation w/ Course**					
L mPFC, dACC, caudate, insula, putamen, thalamus	-8	34	22	4.63	1228
R mPFC, caudate, putamen	6	34	22	4.12	385
L cerebellum	-42	-64	-30	4.42	204
R cerebellum	34	-68	-34	4.33	164

Note

*Course is based on an item adapted from the Structured Interview of Anorexia and Bulimia. Individuals are asked to characterize the course of their disorder from the point at which they were first diagnosed with an eating disorder to the period in which they no longer were diagnosed with an eating disorder. Descriptions of four options for disease course were aided with graphic depictions (S1 Fig).

R = right hemisphere; L = left hemisphere; mPFC = medial prefrontal cortex; dACC = dorsal anterior cingulate cortex

## Discussion

In this study, we sought to characterize the attitudinal and neural responses to social stimuli in weight-restored adults with anorexia nervosa (AN-WR) as compared to a group of healthy controls. First, our results revealed that AN-WR exhibited atypical judgements of attractiveness in regard to weight status relative to controls. Whereas controls exhibited a positive association between increasing weight and increasing attractiveness, this relationship was negative in the AN-WR group. Further interrogation of this association revealed that participants with AN-WR rated individuals at the low end of the normal weight range as more attractive than HCs, but not emaciated individuals. In a disorder in which the sight of emaciated individuals can trigger the urge to intensify weight-loss behaviors, this shift demonstrates progress towards more typical valuation patterns but a persisting bias towards viewing lower weight individuals as more attractive, a bias which may pose vulnerability for relapse.

Our second significant finding was that both in group comparisons, there was reduced activation in the dorsal striatum, the right caudate, in AN-WR relative to HC when viewing full bodies relative to scrambled images. The dorsal striatum has been implicated in mapping response-outcome contingencies and social reward processing [53]. Notably, contrary to hypotheses, group level differences in region of interest analyses were only found for full body images and not for images of smiling faces. Thus, the decreased activation evidenced in the right caudate in AN-WR could reflect the diminished flexibility in mapping outcomes in social contexts. Prior research exploring the nature of reward and learning in AN has documented increased striatal responses to punishments [29] and enhanced self-regulatory processes that may dampen the experience of reward [28] combined with a style that employs less flexible responding in adapting behavior to changing contexts [34]. When considered in social contexts, which necessitate complex and flexible adaptations to changing social circumstances–sometimes in conflictual circumstances, these patterns may reduce social proficiency and consequently the likelihood of experiencing reward from social interactions. Both the dorsal and ventral striatum have been shown to become more activated during mutual cooperation [54]. Thus, one hypothesis regarding striatal difference in AN-WRs is that deficits in social proficiency contribute to diminished reward activation in AN. Neural responses to social rewards may depend, at least in part, on social proficiency. AN has been associated with lower scores on measures of empathy [55], impoverished social networks [56], reduced intimate secure attachments [57], and self-perceptions of impaired social skills [58]. Reduced striatal activation to bodies in AN-WR could reflect reduced sensitivity to rewarding properties of social images, explained by a lack of experience with some of the positive traits of relationships (e.g., bestowing and receiving warmth and comfort).

Notably, the more sustained the disorder course, the greater the decrease in activation, not only in the caudate, but also in more ventral regions of the striatum, including the nucleus accumbens. Thus, a second hypothesis regarding decreased striatal activation is that social images with bodies are provocative of eating disorder symptoms: the drive for thinness inherent in the disorder interferes with the ability to experience the bodies of others as rewarding. Notably, A relentless drive for thinness is a core diagnostic feature of AN. However, this drive for thinness is not just in reference to oneself but also in reference to others. Maladaptive social comparison processes [59], competition [60], and even contagion within specialized eating disorder programs [61] have been reported as individuals with anorexia view other thin individuals as “triggering” and thus threatening to their health and status in regard to low body weight. Thus, it is possible that body images are experienced as less rewarding because of these maladaptive and distancing self-comparison processes that may become more entrenched the more stable the disorder course. It is notable that these differences were not found in relationship to viewing smiling faces, which are relatively innocuous social stimuli in relation to this domain. This raises the interesting possibility that treatment modalities may be able to exploit current technological advances (e.g., texting, use of virtual avatars) to increase social engagement and social proficiency in AN by facilitating engagement with others without body-related cues.

AN has often been described as a disorder of self-awareness and aberrant self-attunement, a consequence, perhaps, of guiding behavior by rules rather than by responding to internal motivational states [62]. Consistent with studies by Via et al. [30] and McAdams et al. [37], we found decreased mPFC activation; however, in our study decreased mPFC activation was negatively correlated with a sustained disorder course. The mPFC has been associated with emotional processing of social stimuli as well as self-referential/self-identity processes. The process of engaging socially is aided by the capacity to sense and label our own emotional and related motivational experiences. With prolonged starvation and dampening of felt experiences via cognitive strategies, changes in the assigned value of stimuli over the course of illness may result from emotional suppression of both positive and negative emotional experiences [63, 64],degradation of emotional regulatory capacities as the illness progresses [65],and illness onset at a key developmental period for the contextualization of visceral experience [19]. Thus, starvation combined with cognitive processes [27] dampens these felt experiences and may lead to diminished self-awareness over time, with potential consequences of diminished social proficiency and related experiences of social reward [66]. Contrary to hypotheses, the vmPFC was not differentially activated in AN-WR relative to controls in the region of interest analyses only the mPFC in exploratory whole brain analyses. The vmPFC has been implicated in computing a “common neural currency” for reward by translating reward signals from different modalities into a common scale [67]. Notably, the vmPFC has been shown to encode the value of non-canonical rewards such as social images [68] and positive emotions [69]. We had hypothesized that starvation during a key period of brain development (adolescence) would result in decreased experience of reward as reflected in the vmPFC. It is possible that the nature of our task was not sensitive enough to detect subtle differences that a more nuanced reward learning task would have captured. It is also possible that with weight restoration, particularly when such restoration has occurred for at least a year (as with the current sample), that the experience of the emotional and sensory features of social reward normalize, while action outcome contingencies in social contexts remain impaired. Finally, it is possible that the severity of starvation itself, as reflected in lowest BMI would be a more relevant parameter to examine more ventral-mediated changes.

In exploratory whole-brain analyses, relative to AN-WR, HCs showed greater activation in the MFG when viewing bodies as compared to scrambled images. McAdams et al. [37] also reported increased MFG activation in adults weight-restored from AN relative to individuals currently diagnosed with AN or healthy controls when engaging in a self-reflective task as contrasted to a reflected-self task (reflecting on the self from another’s perspective)–an opposite pattern from what is seen in healthy controls. One function reported in association with MFG activation is reappraisal [70]. Greater MFG activation in HCs could reflect increased cognitive control or affect regulation when viewing body images. This interpretation is consistent with data showing that when viewing images of their own bodies, women low in eating disorder psychopathology focus visual attention on their more attractive features whereas women high in eating disorder psychopathology focus visual attention on their less attractive features [71]. Thus, one possibility is that HCs engage in adaptive emotion regulatory strategies when confronted with images of other women in a way that promotes a more positive body image. Given the *normative discontent* proposed to characterize the majority of body appraisals due to unrealistic thin body ideals, such active coping may be necessary to maintain positive body esteem in the face of provocative images even in individuals with a healthy body image [72, 73]. The finding by McAdams et al. [37]of increased recruitment of MFG in AN-WR may have reflected the need to actively employ cognitive reappraisal strategies when considering the self, a pattern not seen in actively disordered individuals and not needed in healthy controls.

An additional explanation for why stimuli depicting full figures elicited reduced reward activation in AN-WR is that these women may conceptualize the stimuli differently. It has been shown that down-regulation of reward anticipation leads to decreased activation in the striatum and increased activation in the MFG [74], suggesting that the MFG modulates responsivity in the striatum when individuals consciously attempt to conceptualize the meaning of anticipated rewards. We observed greater activation in the MFG as well as the striatum to combined bodies and faces in healthy controls, possibly due to attempts to conceptualize body images more positively.

Evidence of deviations in reward experience leads to the hypothesis that dopamine function is awry in individuals with AN [6, 75, 76]. Dopamine has a well-characterized role in reward processing [77] and accumulating evidence documents atypical dopaminergic neurotransmission in AN after a year of weight restoration [6, 75, 76]. Notably, self-report and neurobiological evidence suggests that individuals with AN have *heightened* reward sensitivity: Intrusive sensory and visceral experiences in response to both exteroceptive and interoceptive stimuli [11, 22]- suggesting that rewards may be aversive because they experienced so intensely.

Collectively, these observations suggest a model of the pathophysiology of AN in which individuals experience dopamine-linked reward hypersensitivity as intense affective and visceral responses to experiences that are typically reinforcing [5, 22, 78]. Eating disorder symptoms have been proposed to dampen this visceral reactivity and be negatively reinforcing [11]. One hypothesis is that the self-imposed affective and visceral suppression that occurs during the acute phase of AN could ultimately blunt the experience of reward.

Though our investigation demonstrates that reward disruptions in AN extend into the social domain, several limitations are important to acknowledge. Because we did not include a currently-ill sample, we cannot determine the extent to which our results would replicate or show a larger effect in women currently ill. Our measure of disorder course, while obtained after a detailed interview, was only one item and thus may not capture the full complexity of a disorder course. Furthermore, though our sample sizes are similar to or larger than typical fMRI studies of AN [79], our samples are smaller than many behavioral studies performed in similar populations, potentially resulting in reduced power. This latter problem prevented us from looking at stimuli as a function of body weight due to excessive numbers of comparisons for a small sample. Lastly, because our study only exposed participants to socially rewarding images during scanning, it is not possible to generalize or distinguish our findings from the neural responses to other sources of reward in AN.

Consistent with studies of AN employing non-social rewards [80], our results suggest that AN is associated with disrupted reward valuation. As our sample was weight-restored, this result cannot be attributed to starvation. Moreover, given the relatively low EDE-Q scores of our clinical sample, our AN-WR can at least be considered cognitively improved. Thus, distorted social reward processing in these women reflects a predisposing factor, a disease “scar,” or both. Understanding the nature of this disruption is critical because social functioning has been shown to predict long-term prognosis [81]. Consequently, elucidating the nature of social reward disruptions may inform novel strategies for the treatment and putatively, the prevention, of AN.

## Supporting information

S1 Fig Question regarding illness course. (PDF)

S1 File Supplemental methods. (DOCX)

S1 Table Whole brain activation to image type by group. (DOCX)
