# Supplementary material for: Neurobiology of social reward valuation in adults with a history of anorexia nervosa
Source: PLoS One. 2018 Dec 4;13(12):e0205085. doi: 10.1371/journal.pone.0205085 (PMC6279022; doi:10.1371/journal.pone.0205085)

S1 Fig. Question regarding illness course

Between the age when you first developed an eating disorder and the age you stopped having an eating disorder (if you did stop), which of the following best describes the course of your eating disorder? Don't worry about the specific type of eating disorder. With this question we are just trying to understand the pattern of your eating disorder.

0 = no eating disorder

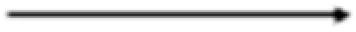

1 = short episodes of eating disorder symptoms followed by longer intervals without eating disorder symptoms. Overall eating disorder symptoms occurred up to 30% of time (or less).

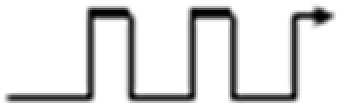

2 = periods of eating disorder symptoms and periods without eating disorder symptoms of approximate equal length. Overall eating disorder symptoms were present about 30-60% of the time.

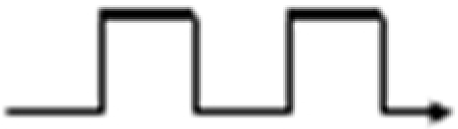

3 = long periods with eating disorder symptoms and only short intervals without episodes of eating disorder symptoms. Eating disorder symptoms were present roughly 60-90% of the time.

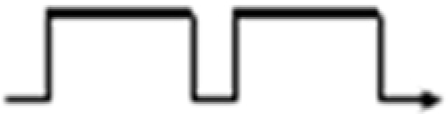

4 = Chronic eating disorder symptoms, almost no intervals without eating disorder symptoms. Eating disorder symptoms were present chronically throughout time, roughly 90-100%. There were no major intervals without eating disorder symptoms.

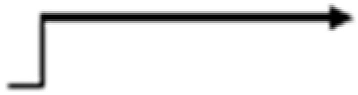

Supplement: S1 Fig — (PDF) [file pone.0205085.s001.pdf]
