# Supplementary material for: Neurobiology of social reward valuation in adults with a history of anorexia nervosa
Source: PLoS One. 2018 Dec 4;13(12):e0205085. doi: 10.1371/journal.pone.0205085 (PMC6279022; doi:10.1371/journal.pone.0205085)
Supplement: S1 File — (DOCX) [file pone.0205085.s002.docx]

**S1 File. Supplemental methods.**

Body images included the face as well as the body from at least the waist up and were cropped and resized to a standard size of 222 x 568. Women in the images wore bathing suits or casual clothing that revealed body shape. Face images were smiling and forward facing, cropped to show the face only, and resized to a standardized size of 568 x 568. We excluded images containing text, depicting celebrities or other recognizable individuals, containing objects other than emotionally neutral background objects, or depicting women who were estimated to be under 18 years of age.

All images were balanced for luminance, and a control image set balanced for low-level visual features was created by phase scrambling the face images. A subset of the face and body images that were not selected for use in the fMRI experiment were displayed to fMRI participants in the mock scanner to familiarize them with the experiment.
