# Supplementary material for: Neurobiology of social reward valuation in adults with a history of anorexia nervosa
Source: PLoS One. 2018 Dec 4;13(12):e0205085. doi: 10.1371/journal.pone.0205085 (PMC6279022; doi:10.1371/journal.pone.0205085)
Supplement: S1 Table — (DOCX) [file pone.0205085.s003.docx]

**S1 Table. Whole brain activation to image type by group**

| **Faces > Scrambled** | | | | | |
| --- | --- | --- | --- | --- | --- |
| **HC** |  |  |  |  |  |
| R occipital/temporal fusiform cortex, R lateral occipital cortex | 28 | -94 | -8 | 5.7 | 4254 |
| Bilateral amygdala, hippocampus, nucleus accumbens, brain stem | 18 | -6 | -16 | 6.14 | 3571 |
| L occipital/temporal fusiform cortex, L lateral occipital cortex | -42 | -84 | -10 | 5.97 | 2652 |
| Cerebellum | -2 | -54 | -34 | 5.11 | 254 |
| L Post-Central Gyrus | -46 | -30 | 58 | 4.1 | 183 |
| Thalamus | 0 | -18 | 6 | 4.1 | 173 |
| **AN-WR** |  |  |  |  |  |
| Bilateral amygdala, hippocampus, lingual gyrus, cuneus, cerebellum | 18 | -6 | -18 | 7.01 | 5781 |
| R occipital/temporal fusiform cortex, R lateral occipital cortex | 42 | -84 | -10 | 5.76 | 4789 |
| L occipital/temporal fusiform cortex, L lateral occipital cortex | -40 | -56 | -18 | 5.81 | 3133 |
| **Bodies > Scrambled** | | | | | |
| **HC** |  |  |  |  |  |
| Thalamus, bilateral amygdala, hippocampus, fusiform gyrus, lateral occipital cortex, lateral orbitofrontal cortex, R superior parietal lobule, striatum, R post- and pre-central gyri | 14 | -34 | 0 | 7.73 | 39067 |
| Cerebellum | 2 | -52 | -38 | 5.82 | 2161 |
| Medial superior frontal gyrus | 4 | 58 | 30 | 5.39 | 1270 |
| Supplementary motor cortex, paracingulate gyrus | 6 | 12 | 52 | 6.01 | 873 |
| L Post-central gyrus | -44 | -30 | 42 | 4.97 | 499 |
| L Pre-central gyrus | -32 | -6 | 62 | 4.07 | 293 |
| L anterior cerebellum | -20 | -38 | -46 | 4.83 | 269 |
| L superior parietal lobule | -28 | -58 | 50 | 4.36 | 228 |
| R anterior cerebellum | 22 | -38 | -46 | 5.08 | 220 |
| **AN-WR** |  |  |  |  |  |
| Bilateral occipital/temporal fusiform cortex, parahippocampal gyrus, bilateral amygdala, brain stem, thalamus | -40 | -54 | -20 | 6.93 | 24013 |
| R inferior/middle frontal gyrus | 48 | 2 | 26 | 4.16 | 645 |
| R post-central gyrus | 46 | -30 | 36 | 5.84 | 561 |
| Superior frontal gyrus | -8 | 58 | 26 | 5.1 | 529 |
| Cerebellum | 0 | -70 | -28 | 4.9 | 495 |
| R superior parietal lobule | 26 | -54 | 48 | 4.19 | 371 |
| L superior parietal lobule | -28 | -56 | 54 | 4.46 | 192 |
| **HC > AN-WR** |  |  |  |  |  |
| R Middle Frontal Gyrus | 46 | 18 | 42 | 4.91 | 183 |

**S1 Table**. **Whole brain activation to image type by group.** HC = healthy controls; AN-WR = anorexia weight-restored; R = right hemisphere; L = left hemisphere
